# Causal inference concepts applied to three observational studies in the context of vaccine development: from theory to practice

**DOI:** 10.1186/s12874-021-01220-1

**Published:** 2021-02-15

**Authors:** Emilia Gvozdenović, Lucio Malvisi, Elisa Cinconze, Stijn Vansteelandt, Phoebe Nakanwagi, Emmanuel Aris, Dominique Rosillon

**Affiliations:** 1 GSK Vaccines, Rue Fleming 2, B-1300 Wavre, Belgium; 2 Present address: Galapagos Pharma, Mechelen, Belgium; 3 GSK Vaccines, Siena, Italy; 4 Ghent University, Ghent, Belgium; 5 London School of Hygiene and Tropical Medicine, London, UK

**Keywords:** Causal inference, Observational studies, Vaccine development, Hill’s criteria, Counterfactual reasoning, Causal diagrams

## Abstract

**Background:**

Randomized controlled trials are considered the gold standard to evaluate causal associations, whereas assessing causality in observational studies is challenging.

**Methods:**

We applied Hill’s Criteria, counterfactual reasoning, and causal diagrams to evaluate a potentially causal relationship between an exposure and outcome in three published observational studies: a) one burden of disease cohort study to determine the association between type 2 diabetes and herpes zoster, b) one post-authorization safety cohort study to assess the effect of AS04-HPV-16/18 vaccine on the risk of autoimmune diseases, and c) one matched case-control study to evaluate the effectiveness of a rotavirus vaccine in preventing hospitalization for rotavirus gastroenteritis.

**Results:**

Among the 9 Hill’s criteria, 8 (Strength, Consistency, Specificity, Temporality, Plausibility, Coherence, Analogy, Experiment) were considered as met for study c, 3 (Temporality, Plausibility, Coherence) for study a, and 2 (Temporary, Plausibility) for study b. For counterfactual reasoning criteria, exchangeability, the most critical assumption, could not be tested. Using these tools, we concluded that causality was very unlikely in study b, unlikely in study a, and very likely in study c. Directed acyclic graphs provided complementary visual structures that identified confounding bias and helped determine the most accurate design and analysis to assess causality.

**Conclusions:**

Based on our assessment we found causal Hill’s criteria and counterfactual thinking valuable in determining some level of certainty about causality in observational studies. Application of causal inference frameworks should be considered in designing and interpreting observational studies.

**Supplementary Information:**

The online version contains supplementary material available at 10.1186/s12874-021-01220-1.

## Background

Since the beginning of this century, an unprecedented amount of scientific research has been conducted on causal inference, the process of assessing causality between exposures and outcomes. Many epidemiological studies are observational in their design, and unlike randomized controlled trials (RCT) where similarity of groups can be experimentally attained, comparability of groups can be difficult or even impossible to demonstrate. This renders causal inference in these cases challenging and conditional on unverifiable assumptions.

To infer causality from association, Sir Austin Bradford Hill synthesized what he called “aspects of association” [1], consisting of 9 distinct criteria that can be used separately or in combination to gather evidence on causal inference. These criteria are known as ‘Hill’s criteria’ and have been extensively used among epidemiologists ever since. More recently, other elaborate frameworks for causal inference have been developed [2–4], stemming from graph theory and counterfactual theories of causation. The counterfactual framework published by Rubin, 1974 [5], led to the definition of three general conditions needed to draw causal inference; exchangeability, consistency and positivity. Causal Diagrams in the form of Directed Acyclic Graphs (DAGs), summarize the assumed relationships between all variables that are relevant to the causal analysis and can be used to detect confounding and selection bias, and to develop insights on how to adjust for them in data analysis or in the study design. We assessed the applicability and limitations of these approaches in drawing causal inference in observational studies in the context of vaccine research.

Vaccine studies have some particularities that may influence causal analyses. For example, vaccination not only protects the vaccinee but can also reduce the transmission of contagious disease in the unvaccinated population (herd immunity). RCTs measure the effect of the vaccine at the individual subject level to demonstrate the vaccine efficacy for approval by health authorities, whereas epidemiological studies are needed to measure the effect of vaccination (effectiveness and impact) at the population level [6]. We assessed how classical and more recent causal inference methods, namely Hill’s criteria, counterfactual framework, and causal diagrams, are applicable in vaccine research, using examples from epidemiological studies.

## Methods

**Fig. 1:**
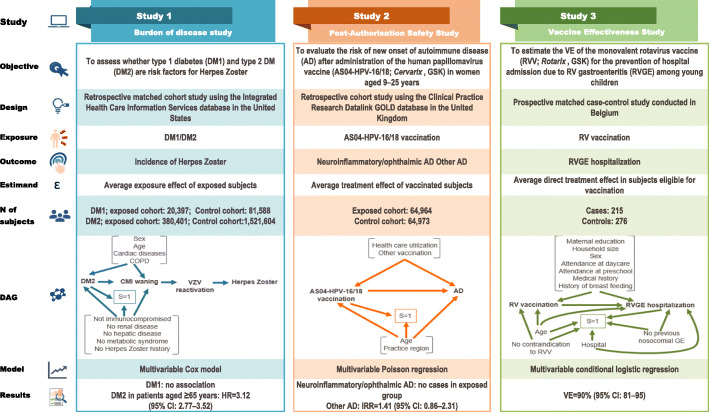
Description of the analyzed studies and causal diagrams. AD: autoimmune disease, IRR: incidence rate ratio, DAG: Directed Acyclic Graph, CI: confidence interval, CMI, cell-mediated immunity, Ctrl: control, DM1/DM2: type 1/type 2 diabetes mellitus, COPD: chronic obstructive pulmonary disease, GE: gastroenteritis, HPV: human papillomavirus, HR: hazard ratio, HZ: herpes zoster, RV: rotavirus, VE: vaccine effectiveness, VZV: varicella zoster virus. Study 1 [8]. DAG: The causal association between DM2 and the risk of HZ was controlled for other factors that might be associated with risk, such as age and underlying cardiac disease and COPD. The square around S = 1 indicates that the analysis is conditional on having been selected into the study. Individuals with renal disease, hepatic disease, metabolic syndrome, history of HZ, or with immunocompromising conditions were excluded from enrolment. The exposure and other factors are associated with the outcome (HZ) via two mediators; waning CMI and VZV reactivation. Study 2 [9]. DAG: S = 1 refers to the selection criteria applied during the study. The square around S = 1 indicates that the analysis is conditional on having been selected into the matched cohorts, age and practice region being the matching factors. The analysis was adjusted for the potential confounders “use of health care resources” and “other vaccinations”. Study 3 [10]. DAG: The square around S = 1 indicates that the analysis is conditional on having been selected into the case-control study. Controls were matched to cases for age and hospital and subjects with previous nosocomial GE or contraindications to RV vaccination were excluded. The analysis was controlled for potential confounders (maternal education, household size, sex, etc.)

We applied Hill’s Criteria, counterfactual reasoning, and causal diagrams to three published studies conducted by GSK: one burden of vaccine target disease (BoD) study, one post-authorization safety study (PASS), and one vaccine effectiveness (VE) study. Based on the published results, for each study Hill’s criteria and counterfactual reasoning were rigorously discussed and assessed by the authors in several meetings until a consensus was reached. No additional analysis was performed during this exercise. Causal diagrams were drawn to depict the exposure, outcome and other factors for each study using standard symbols [7]. The selection and matching factors were symbolized as factors associated with S = 1 [Selection]. Other covariates controlled in the analysis were depicted as associated with both exposure and outcome (Fig. 1).

### Description of studies

The objectives, design and characteristics (estimand, sample size, statistical model, and the main results) of the three selected studies are summarized in Fig. 1.

#### BoD study

This retrospective matched cohort study used the Integrated Health Care Information Services database in the United States (US) to assess whether diabetes mellitus (DM) is a risk factor for herpes zoster (HZ) [8]. Four cohorts were defined: a type I diabetes mellitus cohort (DM1), a type II diabetes mellitus cohort (DM2) and two comparative matched cohorts of non-diabetics. Therefore, the target estimand was the average exposure effect in the exposed subjects. Cox proportional-hazards regression analysis was applied to compare the risk of HZ among diabetics vs. non-diabetics, controlling for other factors that might be associated with the risk of HZ, such as age, sex, cardiac and chronic pulmonary diseases. As shown in the causal diagram (Fig. 1), these factors are also associated with the outcome (HZ) via two mediators, waning immunity and varicella zoster virus (VZV) reactivation. Additionally, several conditions possibly associated with both DM2 and waning immunity were excluded by design (S = 1 refers to selection criteria).

The study showed that DM2 was associated with an increased risk of developing HZ, with age acting as an effect modifier. The Hazard Ratio (HR) associated with DM2 was 3.12 (95% confidence interval [CI]: 2.77–3.52) in subjects aged ≥65 years. Cardiac disease and chronic pulmonary disease were also associated with an increased risk (HR 1.92, 95% CI 1.73–2.13 and 1.52, 95% CI 1.38–1.67, respectively) and modified the association between DM2 and the risk of HZ.

#### PASS: cohort study

This retrospective cohort study used the Clinical Practice Research Datalink GOLD database [11], containing linked anonymized longitudinal medical records from primary care practices in the United Kingdom, to evaluate the risk of new onset of autoimmune disease (AD) in women aged 9–25 years after administration of the human papillomavirus vaccine, AS04-HPV-16/18 (*Cervarix*, GSK) [9]. The two co-primary objectives assessed whether AS04-HPV-16/18 vaccination was associated with an increased risk of neuro-inflammatory/ophthalmic AD or other ADs within 12 months of receiving the first dose. The study included an exposed cohort vaccinated with AS04-HPV-16/18 and an historical unexposed cohort of approximately 65,000 subjects each. The cohorts were frequency-matched for age and practice region identifier. The target estimand was the treatment effect in vaccinated subjects.

The cohorts were compared using a Poisson regression adjusted for potential confounders, “use of health care resources” and “other vaccinations” as depicted in the causal diagram (Fig. 1).

This study did not show evidence of an increased risk of the two co-primary endpoints after vaccination. Specifically, no confirmed cases of neuro-inflammatory/ophthalmic AD were reported in the exposed cohort. For other ADs, the adjusted Incidence Rate Ratio (IRR) for the vaccinated versus the unvaccinated cohort was 1.41 (95% CI: 0.86–2.31).

#### VE study

This prospective, hospital-based, multicenter, matched case-control study assessed the effectiveness of the oral live-attenuated human rotavirus vaccine (HRV; *Rotarix*, GSK) in preventing hospital admissions due to polymerase chain reaction-confirmed rotavirus gastroenteritis among young children in Belgium [10]. The study included 215 children hospitalized with rotavirus gastroenteritis and 276 age and hospital-matched controls. The target estimand was defined as the average direct treatment effect in subjects eligible for vaccination. VE was estimated using a conditional logistic regression model controlling for factors (maternal education, household size, sex, etc.) potentially associated with vaccination and rotavirus disease as shown in the causal diagram (Fig. 1). VE of two doses of HRV was 90% (95% CI: 81–95), and VE of at least one dose was 91% (95% CI: 82–95). VE estimates adjusted for potential confounders were in a similar range.

### Hill’s criteria

For each study, we considered each of the 9 criteria: strength, consistency, specificity, temporality, biological gradient, plausibility, coherence, experiment and analogy. A detailed description of the Hill’s criteria is provided in the Additional file 1.

### Counterfactual reasoning

We assessed the applicability of three components of counterfactual reasoning, namely exchangeability, positivity and consistency, briefly described below (see Additional file 2 for a more detailed description of these criteria).

Exchangeability means that the risk of an outcome in one group (e.g., exposed) would have been the same as the risk of outcome in the other group (e.g., unexposed) of individuals with the same adjustment characteristics, had the individuals in both groups received the same treatment; either the treatment given to those in the exposed group or the treatment given to those in the unexposed group. In randomized controlled trials exchangeability is assumed by design. In observational studies only conditional exchangeability might be reached by controlling for covariates.

Positivity states that no individual, based on his/her adjustment characteristics, is prevented from being either exposed or unexposed. Positivity is required when exposure groups are to be compared: that is, to assess the effect of a treatment on an outcome, individuals are typically assigned to a treatment group or to a control group so that the effect in the two groups can be compared and an effect estimate can be computed. If all individuals in a study (or a subgroup of individuals with the same adjustment characteristics) were assigned to the same group obtaining an effect estimate would not be possible without invoking untestable modelling assumptions.

The concept of consistency is related to the counterfactual notion of setting the exposure status to ‘exposed’ or ‘unexposed’ by some intervention. The consistency assumption states that the obtained conclusions are only relevant for interventions that are ‘non-invasive’ in the sense of doing nothing more than setting the exposure to a particular level. The intervention should define the exposure status and nothing else. The intervention that sets the exposure status to ‘exposed’ should thus not change the outcome of the exposed individuals, if applied to them, and likewise for the unexposed.

## Results

The applicability of the three major components of causal inference are described below and in Table 1 and in Fig. 1.

**Table 1:** Assessment of applicability of causal inference concepts to vaccine studies

Reference	Objective	Design / statistical model	Results	Hill’s Criteria	Counterfactual Thinking
Guignard et al., 2014 [8]	To assess whether diabetes is a risk factor for HZ	Matched cohort study in a US insurance claim database. Cox Proportional hazards regression Analysis adjusted for age was applied to estimate the HR of developing HZ, including age, gender and co-morbidities as covariates	• Type II diabetes in subjects ≥65 years HR 3.12; 95% CI 2.77–3.52 • Type II diabetes in subjects 40–64 years HR 1.51; 95% CI 1.42–1.61 Cardiac disease and chronic pulmonary disease were also risk factors in non-diabetic subjects: • Cardiac disease HR 1.92; 95% CI 1.73–2.13 • Chronic pulmonary disease HR 1.52; 95% CI 1.38–1.67	Str: N Cons: N Spe: N Temp: Y Biogr: N Plau: Y Coh: Y Exp: N Ana: N	Exch: N Pos: Y Cons: N
Willame et al., 2016 [9]	Risk of new onset autoimmune disease in 9–25-year-old women exposed to AS04-HPV-16/18 vaccine in the United Kingdom	Retrospective cohort study to assess the risk of autoimmune diseases in vaccinated and unvaccinated individuals (frequency matched for age and practice region) using a Poisson regression model	• IRR for “other autoimmune diseases” in the vaccinated and unvaccinated cohorts 1.41; 95% CI 0.86–2.31	Str: N Cons: N Spe: N Temp: Y Biogr: N Plau: Y Coh: N Exp: N Ana: N	Exch: N Pos: Y Cons: Y
Braeckman et al., 2012 [10]	Effectiveness of two doses of HRV in preventing hospitalization for rotavirus gastroenteritis in Belgium among children born after October 2006 and aged at least 14 weeks	Prospective, hospital-based, multicenter, matched case-control study. Percent VE = (1- OR)*100. OR calculated from a conditional logistic regression with 95% CI	• Unadjusted VE for two doses 90% (95% CI: 81–95). • Unadjusted VE of at least one dose 91% (95% CI: 82–95)	Str: Y Cons: Y Spe: Y Temp: Y Biogr: N Plau: Y Coh: Y Exp: Y Ana: Y	Conditional Exch: likely Pos: Y Cons: Y

AS04-HPV-16/18 vaccine, *Cervarix*, GSK; *CI* Confidence Interval, *HZ* Herpes zoster, *HR* Hazard ratio, *HRV* Oral human live-attenuated rotavirus vaccine, *IRR* Incidence rate ratio, *OR* Odds ratio, *VE* Vaccine effectiveness, *Str* Strength, *Cons* Consistency, *Spe* Specificity, *Temp* Temporality, *Biogr* Biological Gradient, *Plau* Plausibility, *Coh* Coherence, *EXP* Experiment, *Ana* Analogy, *Exch* Exchangeability, *Pos* Positivity

### BoD study

#### Hill’s criteria

Overall, the association between DM2 and HZ was not considered to be strong because it was only observed in subjects aged ≥65 years. Consistency was not met because several epidemiological studies investigating whether diabetes is a risk factor for HZ have yielded contradictory conclusions, with some studies suggesting that diabetes is a risk factor for HZ [12, 13], and others did not show a statistically significant association [14]. The specificity criterion was not met because there are multiple possible causes of decreased immunity that can reactivate latent VZV leading to the manifestation of HZ [15]. Temporality was met by design because the outcome was incident cases of HZ after a diagnosis of diabetes. The biological gradient was not assessed because of the absence of evaluation of a dose-response relationship between DM2 and HZ. The criterion of plausibility was met because there is scientific evidence showing significantly lower levels of cell-mediated immunity to VZV among patients with diabetes mellitus compared to healthy individuals, suggesting that the increased risk of HZ among diabetics may be related to decreased levels of VZV-specific immunity [15]. Similarly, coherence can also be considered as met because of no conflict with the current knowledge about the natural history of HZ [16]. The experimental criterion was not met because the exposure could not have been induced experimentally in subjects and has therefore never been studied under this study design. The analogy criterion cannot be assessed because there was no other similar exposure.

#### Counterfactual reasoning

Exchangeability was likely not met because there was clearly a difference between diabetic and non-diabetic subjects in terms of other factors such as age and other confounders. Conditional exchangeability was considered plausible after adjustment for these confounding factors, but cannot be guaranteed.

Positivity can be assumed because there are both diabetic and non-diabetic subjects in all levels of covariates included in the model.

The consistency assumption is difficult to evaluate because the exposure is not an intervention, but rather a comorbidity not caused by a well-defined intervention.

### PASS: cohort study

#### Hill’s criteria

The association between vaccine exposure and ADs was not considered to be strong because of the absence of confirmed cases of neuroinflammatory/ophthalmic AD and the IRR lower than 1.5 (and non-statistically significant) for the other ADs. Consistency was not met because five other studies investigating associations between HPV vaccine exposure (AS04-HPV-16/18) and ADs found no evidence of an association [17–21]. Since there are numerous possible causes of ADs it would not be appropriate to conclude that there was specificity. Temporality was met because vaccine administration occurred before the outcome. In terms of biological gradient, the assessment was not feasible as no analyses were conducted that compared the effect of different doses of vaccine on the risk of the outcomes of interest. The plausibility criterion was met as there are known biological mechanisms that could link exposure to vaccine, especially those containing adjuvants, with neuroinflammatory/ophthalmic ADs or other ADs [22]. In addition, as existing scientific knowledge does not support an association between exposure to AS04-HPV-16/18 and ADs, it is reasonable to postulate that coherence was not met. The experiment condition was not met because RCTs showed no differences in the proportion and frequency of ADs between vaccine and control groups [23]. The analogy criterion was not met because no evidence of an association was found for another HPV vaccine [24].

#### Counterfactual reasoning

The subjects in the exposed group and unexposed female group were frequency matched for age and practice region. In addition, the study collected data on other variables such as previous vaccinations and use of health care resources. Matching and controlling for possible confounders supported conditional exchangeability. However, it is likely that the presence of other measured and unmeasured confounders potentially associated with the exposure and the outcome within the sub-cohorts created by matching invalidates conditional exchangeability. Exchangeability was also violated because the exposed and unexposed cohorts were not concurrent.

Although the unexposed female cohort was a historical cohort enrolled before introduction of vaccination, positivity is not violated because there was no adjustment for calendar year. Such adjustment would make exchangeability more plausible but would induce a violation of positivity because there are no exposed and unexposed individuals in the same calendar year.

Consistency is met because vaccination is a well-defined intervention.

### VE study

#### Hill’s criteria

The association between HRV vaccination and prevention of hospital admission for rotavirus gastroenteritis was strong, with an adjusted Odds Ratio of 0.10 (95% CI: 0.05–0.21). Consistency holds because VE has been reported in many other studies in different countries [25]. Specificity criterion can be considered as met because rotavirus vaccines are effective at protecting against rotavirus infection, which is the specific cause of rotavirus gastroenteritis. Temporality criterion is also clearly met since the vaccination status of cases and controls is derived considering only vaccine doses administered at least 14 days before the onset date of gastroenteritis. Since the viral titer in the vaccine is defined, the biological gradient criterion could not be assessed and it is therefore unknown. The biological plausibility of the hypothesis of the causal association between vaccination and disease prevention is supported by the immunological principle that vaccination prevents infections by pathogens, either viruses, bacteria or parasites by eliciting a specific immune response. The coherence of the association is met given that the hypothetical cause-and-effect interpretation does not conflict with the natural history and biology of rotavirus gastroenteritis. The two globally available rotavirus vaccines (HRV and human-bovine rotavirus vaccine) were both shown to be highly efficacious for the prevention of rotavirus gastroenteritis [26]. In agreement with this, the World Health Organization recommends the inclusion of the rotavirus vaccines into all national immunization programs since 2009 [27]. The findings of this study were in line with results of RCT [26, 28], and of ecological studies reporting reductions in the number of admissions attributable to rotavirus after introduction of vaccination [29, 30]. The experiment criterion can therefore be considered as met. Analogy is supported by similar estimates of VE of the human-bovine reassortant rotavirus vaccine observed in case-control studies undertaken in the US [31, 32].

#### Counterfactual reasoning

Controls and cases were matched by date of birth and hospital minimizing the confounding bias by these factors. However, there were differences in some demographic and socioeconomic variables between cases and controls which potentially affect the VE estimate. To control some of these, multivariable analyses were performed, but estimates of adjusted VE accounting for those differences were not significantly different to those obtained in the primary unadjusted analysis.

After taking into account the variables in the analyses as mentioned above, we expect that the set of those covariates are sufficient to achieve conditional exchangeability.

In this study, the conditions of positivity and consistency are met because of presence of vaccinated and unvaccinated in all levels of the covariates in the model, and well-defined vaccination.

## Discussion

We assessed the applicability of Hill’s criteria, counterfactual reasoning to investigate the plausibility of causality in three observational vaccine studies. In addition, we described the relationship between exposure, outcome and other factors using causal diagrams. Based on these assessments, none of the studies allowed us to formally demonstrate causality. Nevertheless, we did conclude that a causal association in the VE study was very likely, based on satisfying 8 out of 9 Hill’s criteria and demonstration of some degree of exchangeability attained due to controlling for known confounders.

For the BoD study, we concluded that a causal association between DM2 and HZ was unlikely. Three out of 9 Hill’s criteria were met: these were temporality, a critical criterion for which the exposure precedes the outcome*,* plausibility and coherence. However, although the consistency criterion was considered as not met, several studies showed an association between DM2 and HZ risk. Similarly, although the strength criterion was reported as not met, an association was found for individuals in subjects aged ≥65 years. Regarding counterfactual reasoning, while positivity could hold, consistency was not met because DM is not related to any intervention. Lastly, exchangeability was unlikely to hold because of differences in the demographic features of the different groups.

For the PASS cohort study, a causal association between HPV vaccination and AD was very unlikely. Only two Hill’s criteria were met (temporality and plausibility*)*. As for the consistency criterion - considered as not met-, it is worth mentioning that, in contrast to the BoD study, an association between HPV vaccination and the risk of AD has not been observed in any other studies. In terms of counterfactual reasoning, vaccination met the consistency assumption. Positivity was met because there was no adjustment for calendar year, but this in turn makes exchangeability unlikely. To mitigate the potential effect of different calendar years for the two female cohorts, the authors of the study included male cohorts to confirm that no adjustment for calendar year was needed. This adds support to a causal interpretation, but only under the unverifiable assumption that what is observed in male cohorts is applicable to the female cohort.

Hill’s criteria [33–37] and causal diagrams [38, 39] have been used previously in the design and interpretation of observational studies, whereas evaluation of the assumptions referred to as identifiability conditions by Hernan and Robins [2] in the counterfactual framework is often done indirectly in the discussion on confounding as part of the study limitations in observational studies. Interestingly counterfactual reasoning introduced the main assumption of exchangeability which was not reflected in Hill’s criteria. The originality of our approach is that we combined all three components and found flaws in all three methods in their applicability to real-life examples of observational studies in the context of vaccine research.

At the time of Hill’s publication 50 years ago, his criteria were not meant to be used be used as a rigid checklist of evidence for causation. However, they have often been used as such. Moreover, their interpretation has changed over time as a result of major advancements in several scientific disciplines, analytical tools and access to big data [40]. As a consequence, some authors have called for revisions to Hill’s criteria [41, 42]. Several criteria are subject to interpretation, for instance the “experiment” criterion, or are difficult to differentiate from each other, such as “plausibility” and “coherence” [43, 44]. In addition, not all Hill’s criteria are applicable or quantifiable for each study type. For example, the “biological gradient” is usually not assessable in vaccine studies because the vaccine dose is fixed. Also, the criterion *“*experiment: removal of the exposure*”* is rarely applicable in observational vaccine studies. Some researchers have recommended an evaluation of confounding factors in addition to Hill’s criteria [45]. A good understanding of what factors cause confounding or selection bias can be difficult in complex (possibly longitudinal) designs. Therefore, causal diagrams can be helpful to gain insight via visual depictions of the relationships between factors, along with graphical tools to assess bias. In addition, counterfactual reasoning provides a formal framework for integrating this notion of confounding in the empirical assessment of causality.

With respect to counterfactual criteria, exchangeability is plausibly attained in intention-to-treat analysis of randomized controlled trials, but generally untestable in observational studies. However, some designs can add support to exchangeability in observational studies, such as self-controlled case-series where the subject is his/her own control. Positivity criteria are met within observational study designs where, within each subgroup defined by the adjustment factors, there is a positive probability of observing exposed as well as unexposed subjects. Consistency is usually met when exposure is a well-defined intervention such as vaccination. However, in many observational studies, exposure is not an intervention but a condition, such as body mass index, or as in our example, DM2. Since these exposures have no immediate connection to a well-defined intervention, consistency as defined in counterfactual reasoning is not applicable. The implication is that even if the effect inferred from such study was causal, caution is needed when interpreting such an effect (e.g. the effect of diabetes) as there is no clearly defined strategy that would e.g. cause or prevent diabetes. Moreover, subjects exposed (or unexposed) could be extracted from large databases with coded data, as was done in our BoD study where DM was defined using International Classification of Diseases (ICD) codes. A systematic review of case definitions for diabetes using ICD-9 and ICD-10 codes showed that coding variations and institutional practices for medical record data extraction could significantly alter the performance of different case definitions used in observational studies [46]. In addition to exchangeability, positivity and consistency, several authors recommend other conditions. Rubin’s Stable Unit-Treatment-Value Assumption (SUTVA) includes the assumption of no interference [47]. In the VE study, the validity of this assumption could be in doubt because the unvaccinated subjects can benefit from an indirect effect of vaccination via herd immunity. As a consequence, the estimand of that study was the direct VE and not the total VE [6].

We used causal diagrams to describe the design of studies, including all known confounders that were considered for adjustment. These diagrams visualize the assumptions made; unknown confounders and known but unmeasurable confounders were therefore not depicted. In observational studies, both exposure and outcome may be measured with error. Since no measurement error corrections could be made, measurement error was not incorporated in our causal diagrams.

Several publications have described the basic principles and recommendations for drawing causal diagrams [48]. However, there is still a lack of agreement in how to depict some observational study designs, such as matched case-control designs [49, 50]. Despite some proposals to depict effect modifiers in causal diagrams [51, 52], the fact that effect modification is scale-dependent implies that effect modifiers cannot be successfully incorporated into causal diagrams, which are designed to be model-free. In spite of these limitations, causal diagrams are a useful tool for identifying variables that should be measured and controlled for at study design [48, 53], and for interpreting the results of analysis [39].

In the three selected studies, we assessed the causality between a single exposure and a disease: DM2 and HZ risk, HRV vaccination and hospitalization for rotavirus gastroenteritis, and AS04-HPV-16/18 vaccination and AD. In this context, it is worth pointing out that the etiology of most diseases is multi-factorial. For instance, decreased levels of VZV-specific immunity are known to lead to the reactivation of the latent VZV that subsequently results in the clinical manifestation of HZ [8]. However, there are numerous causes of waning immunity including advancing age, comorbidities, immunosuppressive treatments, etc. Thus, even in the case of a well-defined medical condition and biological cause, a set of complex contributing mechanisms and interactions can occur. In contrast, the PASS study included a composite endpoint (19 different diseases were defined) and the etiology of many ADs is only partially elucidated. Moreover, many or most ADs are a result of multiple factors that can interact or co-exist, including genetic predisposition and potential environmental triggers [43].

Observational studies never account for all possible factors that lead to an outcome; there are always unknown factors or unmeasurable known factors which can be confounders. By comparison, in RCT the unknown or unmeasurable factors are, by design, expected to be consistently distributed in the treated and control groups.

Our analysis was intentionally limited to assessing how causal inference approaches can be applied to observational studies in the context of vaccines. We reached a consensus to include three studies assessing different objectives frequently addressed in vaccine clinical research; (vaccine effectiveness, burden of disease and safety) which we considered to provide acceptable diversity in our examples. The objective was not an exhaustive review of the various causal inference methods or of recent developments in causal inference and analytical methods such as Inverse probability weighting, G-formula, G-estimation, Instrumental variable estimation, and so on [54]. This will be the next step of our research using both real data and simulations.

Our review was limited to three study designs. Nevertheless, our analysis can be used to guide observational study design in the context of causal inference. Assessment of potential causal effects using real data should start by depicting the existing scientific knowledge about a clearly defined research question. Exposure, outcome and confounders need to be explicitly defined and causal diagrams developed to visualize the relationship between these three major elements including the unmeasured confounders. Conditional exchangeability could be reached provided that the confounders are controlled either by study design or by adjustment of the effect estimate during the analysis. Standard methods to assess causality in observational studies (for example, propensity score-based methods, inverse probability of treatment weighting, multivariable regression, etc.) require the assumption of no unmeasured confounding. In our studies, we did not consider possible unmeasured confounders but these can occur in real-world data and therefore need to be addressed. Methods have been developed to control for them [55], but these require alternative assumptions for identification (e.g., that certain measured variables are so-called ‘instruments’). Interestingly, a recent revision of the European Medicines Agency pharmacoepidemiology guidance provides extensive recommendations to address confounding [56]. Finally, standard statistical software (for example SAS or Stata) now include procedures for applying different causal inference methods, which prevent the extrapolation bias to which standard regression methods are prone [57].

## Conclusion

In conclusion, the application of Hill’s criteria and counterfactual reasoning in three observational studies showed that definite causality could not be demonstrated. However, these approaches allowed us to determine the level of certainty of causality, in each study and evaluating it from very unlikely to very likely. The causal diagrams appeared to be a complementary tool which can help in designing observational studies and interpreting results of analysis. Even if a causal effect could not be demonstrated using only observational data, these studies have real value in decision making in public health, being complementary to RCTs which are not always feasible or ethical. Moreover, major scientific advances, such as Darwin’s development of the theory of natural selection, were generated entirely from observational data, highlighting the critical contribution of such data to scientific thought.

### Trademark statement

*Cervarix* and *Rotarix* are trademarks owned by or licensed to the GSK group of companies.

## Supplementary Information

**Additional file 1: Table S1.** Hill’s Criteria for causality, 1965

**Additional file 2.** Detailed description of exchangeability, positivity and consistency

## Data Availability

The data from the 3 observational studies analyzed herein were retrieved from the source publications [8–10].

## Abbreviations

AD: Autoimmune disease
Ana: Analogy
AS04-HPV-16/18: AS-04-adjuvanted human papillomavirus vaccine
Biogr: Biological Gradient
BoD: Burden of vaccine target disease
CI: Confidence interval
Coh: Coherence
Cons: Consistency
COPD: Chronic obstructive pulmonary disease
CMI: Cell-mediated immunity
DAG: Directed Acyclic Graph
DM1: Type I diabetes mellitus
DM2: Type II diabetes mellitus
Exch: Exchangeability
EXP: Experiment
GE: Gastroenteritis
HZ: Herpes zoster
HR: Hazard ratio
HRV: Oral human live-attenuated rotavirus vaccine
ICD: International classification of diseases
IRR: Incidence rate ratio
OR: Odds ratio
PASS: Post-authorization safety study
Plau: Plausibility
Pos: Positivity
RCT: Randomized controlled trial
RVV: Rotavirus vaccination
S: Selection
Spe: Specificity
Str: Strength
Temp: Temporality
VE: Vaccine effectiveness
US: United States
VZV: Varicella-zoster virus
