# Supplementary material for: Causal inference concepts applied to three observational studies in the context of vaccine development: from theory to practice
Source: BMC Med Res Methodol. 2021 Feb 15;21:35. doi: 10.1186/s12874-021-01220-1 (PMC7882866; doi:10.1186/s12874-021-01220-1)
Supplement: Supplementary file 1 — Additional file 1: Table S1. Hill’s Criteria for causality, 1965 [file 12874_2021_1220_MOESM1_ESM.docx]

**Additional file 1**

**Table S1** Hill’s Criteria for causality, 1965

| Criterion | Definition |
| --- | --- |
| Strength | Strong associations between exposure and disease are less likely to be due to undetected confounding or bias. However, weak associations do not rule out causal connections. |
| Consistency | Consistent findings observed in several different studies using a variety of populations and study designs strengthen the likelihood of a causal effect. |
| Specificity | Causation is more likely if there is a cause that leads to a single effect, not to multiple effects. |
| Temporality | Essential criterion: temporal sequence of the association, the exposure must precede the outcome. |
| Biological gradient | A causal association can be supported by the presence of a dose–response relationship, for instance an increase in the magnitude of exposure should lead to an increase in the magnitude of the risk of the outcome. |
| Plausibility | A plausible mechanism based on existing biological models explaining the association between the exposure and the outcome is helpful, but is dependent on the current state of scientific knowledge. |
| Coherence | A cause-and effect interpretation for an association should not conflict with what it is known of the natural history and biology of the disease. |
| Experiment | Removal of the exposure leads to a reduction in the risk of the outcome. |
| Analogy | When a causal association between a particular exposure and outcome is well known, it is more likely that an association between a similar exposure and a similar outcome exists as well. |
