# Supplementary material for: Causal inference concepts applied to three observational studies in the context of vaccine development: from theory to practice
Source: BMC Med Res Methodol. 2021 Feb 15;21:35. doi: 10.1186/s12874-021-01220-1 (PMC7882866; doi:10.1186/s12874-021-01220-1)
Supplement: Supplementary file 2 — Additional file 2. Detailed description of exchangeability, positivity and consistency [file 12874_2021_1220_MOESM2_ESM.docx]

**Additional file 2**

**Counterfactual reasoning**

When defining the concept of causal effect for an individual it is helpful to think of two dichotomous variables: treatment variable X (where 1 = treatment and 0 = no treatment) and outcome variable Y (where 1 = disease and 0 = no disease). Two potential scenarios may be observed for a given individual: Y^x=1^, the outcome observed when treatment X=1, and Y^x=0^, the outcome observed when treatment X=0. With these notations in mind it can be concluded that there is a causal effect for an individual when the outcome observed on the treated individual is different from the outcome observed on the same individual under no treatment: Y^x=1^ $\neq$ Y^x=0^, i.e., the risk of disease is different based on whether the individual has been treated or not.

The two variables Y^x=1^ and Y^x=0^ are known as counterfactual outcomes. However, the two counterfactual outcomes can never be simultaneously observed because the same individual cannot simultaneously receive and not receive a treatment. Therefore, causality between an intervention and an outcome cannot be evaluated at the individual level. Similarly, when we expand the counterfactual thinking to a population, as would be the case in a study using real world data, only one outcome can be observed for each individual: the one associated with the assigned treatment group. In such a scenario it is possible to assess the association between a treatment and the outcome since we are measuring the effect of treatment and of no treatment (e.g., through a risk ratio or risk difference) on the outcome in two distinct populations (one population where individuals are treated and one population where individuals are not treated). However, to be able to establish *causation* this assessment should be conducted in the same population; once where all individuals in the population are treated and once where individuals in the same population are not treated. This is typically not feasible the in real world. A study design close to this ideal scenario is the randomized controlled trial, through random assignment of individuals to different treatment groups. The goal of randomization is to achieve comparability between treatment groups in terms of measured and unmeasured factors (potentially associated with exposure and outcome, i.e., confounders). Thus ideally, it would be expected that the probability of an outcome in one group (e.g., group A) would have been the same as the probability of the same outcome in the other group (e.g., group B) if the individuals in group A had received the treatment administered to group B. Consequently, the probability of an outcome in group A would equal the probability of the same outcome in group B had individuals in group B received the treatment given to group A. This condition makes the groups exchangeable with each other and is known as exchangeability. When exchangeability holds, independence between the counterfactual outcome and the observed treatment is achieved. With such conditions, association and causation coincide.

Due to the lack of random treatment assignment and the presence of factors that may confound the relationship between an exposure and an outcome, referring to causal inference in the interpretation of results coming from observational studies is more challenging and may be more prone to criticism. However, observational studies could be treated as conditionally randomized trials when three conditions are met, namely conditional *exchangeability*, *positivity*, and *consistency*.

**Exchangeability**

In a conditionally randomized trial there is an imbalance in the distribution of a third factor Z (associated with the exposure and the outcome) between the treated and the untreated groups. This is possible when the probability of receiving treatment depends on Z, and Z is associated with the treatment and the outcome. Here, subjects with one level of Z may be more likely to receive treatment compared to subjects with the other level of Z (for a dichotomous factor). As a practical example, if treatment is heart transplant vs. no heart transplant and the outcome is survival vs. no survival, subjects in critical conditions (Z=1) may be more likely to receive heart transplant compared to those in non-critical conditions (Z=0). In such a scenario the treated and untreated groups may not be exchangeable. However, they are considered exchangeable within defined boundaries of Z. For instance, when Z=1 the probability of the outcome among treated individuals had they remained untreated would be the same as the probability of the outcome among untreated individuals, and vice versa. As a result, in a conditionally randomized trial there is conditional exchangeability within defined boundaries of Z because it is expected that in each stratum there will be no imbalance in the distribution of other predictors.

In observational studies, treatment is not randomly assigned and it is likely that being exposed to treatment or some risk factor for the outcome is linked to other predictors of the outcome. This results in an unequal distribution of factors potentially associated with the outcome between the exposed and unexposed group (or, equivalently, between the treated and untreated groups). If Z was the only factor with an uneven distribution between the groups being compared, the observational study would be analogous to a conditionally randomized trial, as described above. Nevertheless, in observational studies it is unknown whether Z is the only factor with an unbalanced distribution between groups, and there may be other unmeasured factors associated with the outcome that could affect the probability of receiving treatment, or similarly, of being exposed to a predictor. Thus, exchangeability in observational studies may never be fully met. However, we can enhance our confidence that conditional exchangeability is approximately true by collecting data on relevant variables and by making plausible assumptions based on expert knowledge in the field.

**Positivity**

To assess the effect of a treatment on an outcome in a clinical trial, individuals are typically assigned to a treatment group or to a control group so that the effect in the two groups can be compared, and an effect estimate can be computed. If all individuals in a study were assigned to the same group, i.e., all individuals are assigned to the treatment group or all to the control group, obtaining an effect estimate would not be possible. Thus, to estimate a treatment effect more than one treatment level is required, and the probability of being assigned to each of the possible treatment levels must be >0. This condition is known as positivity. In conditionally randomized trials the probability of being assigned to all foreseen treatment levels within levels of Z is positive by design (i.e., for each category of Z the probability of being assigned to any treatment level must be >0 and <1), by which positivity holds. Additionally, positivity is only required to hold within levels of variables that may confound the association between a predictor X and the outcome Y, and for which exchangeability is required (e.g., Z). In an observational study setting, positivity is not ensured because it is not possible to control these associations to the same extent as in a randomized trial.

**Consistency**

The concept of consistency is related to the potential presence of different types of the same, treatment or intervention in a randomized experiment. For instance, it is possible that surgeons use slightly different surgical techniques or procedures when operating on patients with a common condition, or that dieticians intervene with slightly different approaches to reduce obesity. In these scenarios, the causal effect on the outcome can differ depending on the version of treatment received, and the causal effect in the population is an average of the causal effects reported in each group of individuals who received a particular version of the treatment or intervention. Since the definition of X can vary, the concept of counterfactual outcomes is undermined and referring to counterfactual outcomes and causal effect becomes ambiguous. This unfavorable situation can be substantially reduced in randomized trials by providing all necessary details regarding treatment or intervention procedures in the study protocol, so that causal inferences can be made with more confidence. However, in observational studies it may be more challenging to achieve control over a treatment, intervention, or exposure in the real world, and to reduce the differences in terms of exposure among individuals belonging to the same exposure group. Similar to randomized trials, it is possible to reduce variability in exposure and ambiguousness in causal questions by specifying as many details as necessary, keeping in mind that these elements can never be removed entirely. Rather than aiming to reach complete elimination of exposure variability and to develop a perfect causal question, the goal should be to provide all meaningful details and remove irrelevant details such that the outcome will be a *sufficiently well-defined* version of exposure.[^1^](#_ENREF_1) The meaningfulness of sufficiently well-defined versions of exposure and that of causal questions can be enhanced by increased knowledge and expertise in the topic being investigated. In this way, interpreting counterfactual outcomes can be achieved with higher levels of confidence, and conclusions about causal relationships will be more robust.

**Reference**

1. Hernán MA, Robins JM (2020). Causal Inference: What If. Boca Raton: Chapman & Hall/CRC. .
